# Super-focusing of center-covered engineered microsphere

**DOI:** 10.1038/srep31637

**Published:** 2016-08-16

**Authors:** Mengxue Wu, Rui Chen, Jiahao Soh, Yue Shen, Lishi Jiao, Jianfeng Wu, Xudong Chen, Rong Ji, Minghui Hong

**Affiliations:** 1 Department of Electrical and Computer Engineering, National University of Singapore, 4 Engineering Drive 3, 117576, Singapore; 2 Department of Electronic Engineering, Tsinghua University, Beijing, 100084, China; 3 Department of Physics, National University of Singapore, 2 Sciecne Drive 3, 117542, Singapore; 4 Agency for Science, Technology and Research (A*STAR), 2 Fusionopolis way, 138634, Singapore

## Abstract

Engineered microsphere possesses the advantage of strong light manipulation at sub-wavelength scale and emerges as a promising candidate to shrink the focal spot size. Here we demonstrated a center-covered engineered microsphere which can adjust the transverse component of the incident beam and achieve a sharp photonic nanojet. Modification of the beam width and working distance of the photonic nanojet were achieved by tuning the cover ratio of the engineered microsphere, leading to a sharp spot size which exceeded the optical diffraction limit. At a wavelength of 633 nm, a focal spot of 245 nm (0.387 *λ*) was achieved experimentally under plane wave illumination. Strong localized field with Bessel-like distribution was demonstrated by employing the linearly polarized beam and a center-covered mask being engineered on the microsphere.

Micro-lenses, with a size of a few wavelengths, exhibit excellent abilities to confine incident light and generate small focal spot which exceeds the optical diffraction limit at around half of the incident wavelength. Among which, the most investigated micro-lenses are microspheres and microcylinders. It has been demonstrated early in 2000 by Lu and Luk’aynchuk *et al*.^1^. Later, Chen *et al*. studied the field enhancement at the shadow side of a microcylinder under plane wave illumination and termed it as “photonic nanojet”^2^. Excellent optical properties of the photonic nanojet, such as non-diffracting, strong localized field intensity and sharp focal spot, have proved to be beneficial for various applications: nanoparticle detection, optical nanolithography, and super-resolution imaging. Among which, a small beam waist of the focal spot is the most desired property as it characterizes the converging ability of the microlens and plays a key role in these applications. It is found that when a nanoparticle is located within the focal region of a microsphere, the back-scattering intensity can be greatly enhanced. This enhancement is applied for detecting nanoparticles in liquid and nanoparticles at a size of 20 nm can be identified^3^,^4^. Furthermore, it is concluded that the detection sensitivity can be greatly enhanced when the beam width of the photonic nanojet is small. On the other hand, the photonic nanojet generated by the microsphere is applied as the exposure beam in optical lithography^5^,^6^,^7^,^8^,^9^,^10^, where the minimum line width of the fabricated patterns is directly dominated by the beam width of the photonic nanojet. Therefore, when a sharp photonic nanojet is achieved, the pattern size can be reduced correspondingly. Also, in optical super-resolution imaging, the sample interacts with the electric field of the photonic nanojet, and generates scattered wave which propagates through the microsphere to form the image. When combined with a confocal microscope, resolution of 25 nm in air can be achieved, pushing the super-resolution ability of microspheres to a new limit^11^,^12^.

Approaches to modify the optical properties of the photonic nanojet, including changing the refractive index and diameter of the microspheres, varying the illumination conditions and the shapes of the microspheres, have been proposed^13^,^14^,^15^,^16^,^17^,^18^,^19^. Another effective way to tune the photonic nanojet is fabricating micro-structure on the spherical surface and modify the contribution of different field components to the total field^20^. This approach introduces freedom in designing the surface structure of the microsphere and manipulating the interaction of the microsphere with incident beam. Various functional structures can be fabricated on the microsphere to achieve modification of the beam size and working distance of the photonic nanojet. When linear or circular polarization was illuminated on center-covered focusing lenses, Bessel beam can be generated^21^,^22^. A Bessel-like photonic nanojet was reported by designing a core-shell microsphere and illuminated under linearly polarized beam^23^. Another work which simulated a flat PEC filter located above the microsphere proved the modification ability of the engineered microsphere^24^. The difference in our work is that we employed a blanket-like Platinum (Pt) cover on dielectric microsphere surface and prevented the multiple reflection at the gap between the microsphere top surface and metal boundary. As the total electric field intensity pattern at the cross-section perpendicular to the polarization direction is determined by the transverse field component, we can expect a sharper focal spot when the transverse component is modulated to a smaller spot. The designed mask, which blocks the beam propagating in the vicinity of the optical axis, is important to the formation of a sharp focal spot.

In this paper, a photonic nanojet with FWHM = 245 nm (0.387 *λ*, *λ* = 633 nm) is demonstrated in both simulation and experiment by combining the properties of the linear polarization illumination, center-covered mask, and the dielectric microsphere. Compared with conventional ways of modifying the beam shape of the photonic nanojet, the highlights of our work are as follows. Firstly, we achieve a sharp photonic nanojet using a linearly polarized beam and center-covered mask created on the microsphere. Secondly, unlike conventional large scale mask coated on an objective lens, the cover mask we employed is designed and fabricated directly onto the microsphere surface. By decorating the functional micro/nano-structures, the vector properties of the electric field of the photonic nanojet of the microspheres can be modulated. Most importantly, the beam width of the photonic nanojet breaks the optical diffraction limit. In this work, 3D finite-difference time-domain (Lumerical FDTD) is used for the theoretical analysis and the experimental verifications are carried out using a high resolution optical microscope under the illumination of linear polarization beam.

## Results and Discussion

**Figure 1:**
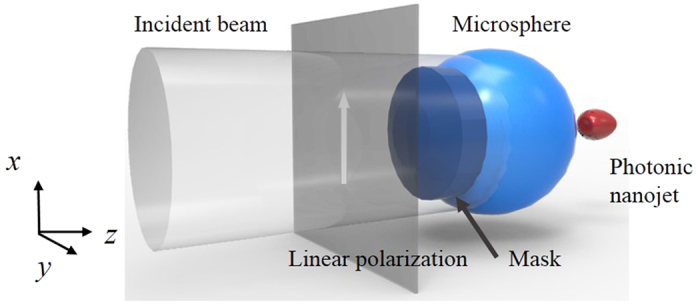
Design of the center-covered engineered microsphere for super-focusing of linear polarized beam. The incident beam is 633 nm and diameter of the microsphere is 10 *μ*m.

Figure 1 schematically shows the focusing of an engineered microsphere illuminated by a linearly polarized beam. The dielectric microsphere has a diameter of 10 *μ*m and refractive index of 1.5. The incident beam is a plane wave with direction indicated by the arrow. The opaque cover on the engineered microsphere surface functions as a filter which removes the beams propagating near the optical axis. This layer is fabricated by depositing Platinum (Pt) with a thickness of 1 *μ*m onto the surface of the microsphere. The electric field at the focal plane of the engineered microsphere can be decomposed into longitudinal (*E*_*z*_) and transverse (*E*_*x*_ and *E*_*y*_) field components. In this paper, we study the *x*-axis polarized plane wave incidence. Therefore, the intensity contribution of the *E*_*y*_ field is ignored as it is of two magnitudes smaller in intensity compared to the *E*_*x*_ field. It should be noted that due to the asymmetric nature of the linear polarization beam, the intensity pattern of the *E*_*z*_ field is two symmetric peaks along the *x*-axis, which results in elongation along the polarization direction. In this paper, we focus our discussion on the *yz* plane, which characterizes the minimum spot size achievable by the design. Modification of the *E*_*x*_ field leads to generation of different photonic nanojets with tunable beam sizes and working distances. When the cover mask is introduced at the center of the microsphere, the light rays propagating in the vicinity of the optical axis is reflected and therefore only the beams which locate farther from the axis are allowed to enter the microsphere and contribute in forming the photonic nanojet. Based on Snell’s law of optical transmission and reflection, incident beam propagating farther from the optical axis focuses near the surface of the microsphere and vice versa. After focused by the microsphere, these beams formed different angles with the optical axis and the intensity of the focal spot is calculated as the integration from the smallest angle to the largest. The contribution to the total field from the incident rays are different. Consider the beam propagating near to the optical axis, after being focused by the microsphere, the angle formed by the transmitted beam and the optical axis is small, which is comparable to the result of a focusing lens with low numerical aperture(NA). At a low NA, the focal spot possesses a long working distance with a large spot size. For the beam propagating further from the optical axis, the focusing phenomenon is similar to the case of a high NA lens. These incident beam converges through the microsphere and focuses at a small working distance with a sharp beam waist. Based on the different contributions of the incident beam to the photonic nanojet, a center-covered mask is designed on the microsphere to allow only the beam propagating farther from the optical axis to form the focal spot. In this way, a sharp photonic nanojet with small working distance can be achieved. To investigate the effect of the opaque cover on the microsphere, the cover ratio is defined as the radius of the deposited Pt divided by the radius of the microsphere (in top view).




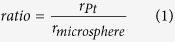




When the cover ratio changes, the amount of the incident beam entering the microsphere is modified. To clarify the effect of different cover ratios, we define the smallest NA introduced by the incident ray propagating through the microsphere as *NA*_*min*_. As the total field intensity is the integral of the beam with different angles to the optical axis, the minimum NA determines the lower boundary of the integral range. When the *NA*_*min*_ is high, contribution from the parasitic beams, which are blocked by the mask, are ignored in the formation of the total field. At large cover ratio, only a thin annular shape beam is focused by the engineered microsphere and interfered along the optical axis.

**Figure 2:**
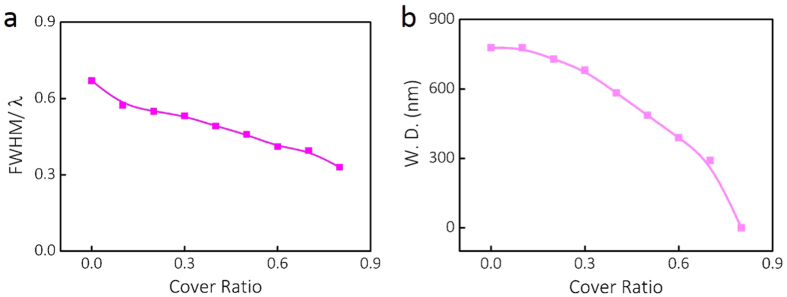
Dependence of focal spot size and working distance of the photonic nanojet on cover ratio. (**a**) Simulated FWHM/*λ* value for different cover ratios. (**b**) Simulated working distance for different cover ratios.

To evaluate the performance of the engineered microsphere under the *x*-axis linearly polarized focused beam, simulations are performed with 3D FDTD. Perfect matching layers are selected as boundary conditions with non-uniform mesh size of *λ*/10. Results are collected from the *yz* plane. The FWHM and working distance (W.D.) values of the photonic nanojet are evaluated at the highest intensity point along the optical axis outside the boundary of the engineered microsphere. Simulation results for the linear polarization illumination are shown in Fig. 2. The cover ratio of the engineered microsphere is changed from 0 to 0.80. In Fig. 2a, the dependance of FWHM on the cover ratio is presented. It can be observed that when the cover ratio increases, the FWHM of the engineered microsphere decreases and achieves a spot size beyond the theoretical diffraction limit of around 0.50 *λ*. Comparing the results, it can be observed that a single microsphere without any decorated cover layer possesses a FWHM of 424.11 nm (0.67 *λ*) under 633 nm illumination wavelength. When the cover ratio increases from 0 to 0.20, corresponding to the mask cover radius of 0 and 1 *μ*m, a modulation of the FWHM from 424.11 nm to 321.84 nm is realized. The difference in FWHM is around 102.27 nm and 0.162 *λ*. At a small cover ratio, the *NA*_*min*_ of the engineered microsphere is low, resulting in a focal spot within the optical diffraction limit. When the cover ratio changes from 0.30 to 0.50, the Pt layer spreads over a half of the microsphere surface, the FWHM is shown to vary from 299.56 nm to 271.37 nm, which changes at 0.045 *λ*. It can be concluded that the engineered microsphere at the cover ratio of 0.30 exhibits the ability to focus the plane wave incident beam to below 0.50 *λ*. This indicates an effective modulation of the photonic nanojet by the engineered microsphere. Further enlarging the cover ratio from 0.60 to 0.80, only a thin ring shape of the incident light can enter the microsphere. At this stage, the incident beam enters the microsphere at a longer distance away from the origin of the optical axis. Thus, the *NA*_*min*_ of the engineered microsphere is high. The decrease of the FWHM value in this region is observed to be from 259.57 nm to 208.33 nm, where a sharp photonic nanojet and high sidelobe are observed. The sharpest focal spot achieved is 0.33 *λ*, corresponding to the cover ratio of 0.80. Compared the FWHM between 0 and 0.80 cover ratio, obvious change from 424.11 nm to 208.33 nm, indicating a strong modification of the beam width of the photonic nanojet. Another important property to characterize the photonic nanojet is the working distance, calculated as the distance between the shadow side of the microsphere boundary and the highest intensity of the photonic nanojet along optical axis. As shown in Fig. 2b, when the cover ratio of the engineered microsphere increases, the W.D. decreases, indicating that the photonic nanojet is close to the microsphere surface. When the cover ratio is 0, the W.D. is calculated as 777.78 nm. Further increase the cover ratio to 0.40, the W.D. dropped to 583.33 nm, which falls within a wavelength of the incident beam. At a higher cover ratio, for example at 0.80, the W.D. is 0 and the highest intensity of the photonic nanojet is located at the shadow side of the engineered microsphere boundary. This indicates that the engineered microsphere can modify both the beam size and the W.D. of the photonic nanojet. As the cover ratio of the engineered microsphere increases, the intensity of the photonic nanojet decreases. When the cover ratio is 0, the highest intensity is 315 times higher compared to the incident beam. The Pt cover limits the amount of transmitting incident beam through the microsphere and leads to low intensity of the photonic nanojet. At a cover ratio of 0.50, the intensity drops to 271 times of magnification of incident beam and at high cover ratio of 0.80, the intensity becomes 199 times.

**Figure 3:**
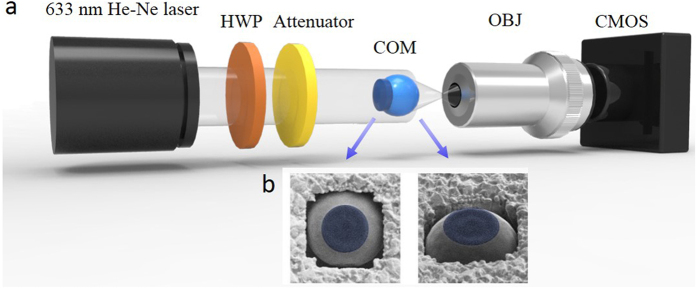
Characterization of the center-covered microsphere focusing. (**a**) Schematic of the experimental setup. Abbreviations for optical components: HWP: half-wave plate. OBJ: objective lens. CMOS: Complementary metal-oxide semiconductor. (**b**) Top and side views of a center-covered microsphere located in a thin gold membrane. Artificial dark blue color to indicate the location of the Pt cover layer.

**Figure 4:**
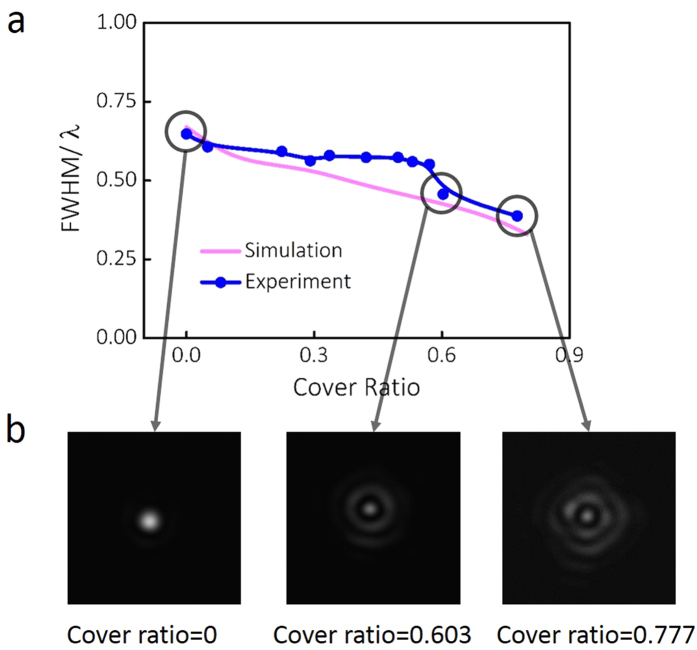
Simulation and experimental results of the beam size for center-covered engineered microspheres at different cover ratios. (**a**) Dependence of the beam width of the photonic nanojet on cover ratio. (**b**) Normalized intensity distribution of the cross-section of the photonic nanojet along the optical axis captured by the CMOS camera. The cover ratio is labelled under each figure and the length of the frames are 5 *μ*m.

To experimentally exam and verify the optical properties of the center-covered engineered microspheres, the focusing characterization of the photonic nanojet is performed with a home-built optical microscope imaging system, as shown in Fig. 3. The optical path of the whole setup is shown in Fig. 3a. The designed engineered microsphere characterized by the scanning electron microscope (SEM) is shown in Fig. 3b, where the microsphere at a diameter of around 10 *μ*m and covered with Pt layer is located within a thin gold membrane. It can be seen that the gold membrane holds the waist of the microsphere and provides strong support during the focus ion beam (FIB) fabrication and optical microscope characterization. Artificial dark blue color is applied to indicate the location of the Pt cover layer. During the experiment, a He-Ne 633 nm laser with linear polarization is employed as the illumination source. After the beam passes through the half wave plate, polarization direction is modulated. The photonic nanojets generated at the shadow side of the microspheres are collected with an 150× objective lens (NA = 0.9) and recorded by a high resolution CMOS camera. The detailed characterization process is described in Methods section. Using this experimental setup, the photonic nanojet of the engineered microsphere is observed and captured. Figure 4 presents the experimental results of the beam size and the cross-section of the photonic nanojet compared to the previous simulation results. As shown in Fig. 4a, engineered microsphere with different cover ratios are placed on the sample stage of the optical microscope in transmission mode. Incident beam illuminates onto the engineered surface of the microsphere and photonic nanojet formed at the shadow side is collected. The cross-section of the highest intensity plane along the optical axis is captured and analyzed. Figure 4a shows the experimental results for measuring the FWHM value at each cover ratio, with the simulated results as a comparison. The cover ratio of the fabricated engineered microsphere is changed from 0 to 0.78, with a linear increase of around 0.13 each step. When the cover ratio is 0, which indicates an original microsphere with no artificial structures, a FWHM of 410 nm is measured in the optical system, corresponding to a value of 0.647 *λ*. This value agrees well with the simulation prediction. It can be observed that with the increase of cover ratio, the beam size of the photonic nanojet decreases. When an engineered microsphere with large cover ratio of 0.603 is applied in the experiment, the FWHM value is measured as 289 nm, which reaches 0.456 *λ*. This indicates that when the cover ratio exceeds 0.603, a super-focusing effect of the engineered microsphere can be achieved in experiment. At a higher cover ratio of 0.777, the FWHM of the photonic nanojet can be reduced to 245 nm, 0.387 *λ*, and the intensity distribution is close to a Bessel beam generated by a high NA optical system. To have a detailed comparison of the focal spot intensity patterns of the engineered microspheres at different cover ratios, the cross-section perpendicular to the optical axis is shown in Fig. 4b. The size of all the square frames is 5 *μ*m. Three important cover ratios are taken as 0, 0.603, and 0.777, which represent the original microsphere without surface engineering, the cover ratio beyond which focal spot size is smaller than 0.5 *λ* and the largest cover ratio fabricated and characterized with the optical system. As it can be seen from Fig. 4b, a microsphere can generate a focal spot with no sidelobe and with the increasing of cover ratio, the sidelobe becomes higher and the intensity distribution is similar to a Bessel beam. The focal spot size at the center of the optical axis is reduced significantly. At high cover ratio, the annular shape incident beam has a large *NA*_*min*_ and the interference at the focal spot is similar to the generation of the Bessel beam. The difference in simulation and experiment could be resulted from difficulty locating the *y*-axis from the intensity patterns on CMOS camera (assume the incident beam propagates along the *z*-axis and the polarization is along *x*-axis). At a higher cover ratio, the different lengths of the electric fields along the *x* and *y* directions become more obvious to be identified. It can be concluded that for linear polarization illumination, the designed center-covered microsphere can function as a focusing lens which generates a sharp focal spot under plane wave illumination. When the cover ratio increases, this phenomenon becomes more obvious as the low *NA*_*min*_ contribution to the total field is filtered out. The W.D. of the photonic nanojet is close to the surface of the microsphere. However, the boundary of the microsphere is hard to be identified under the transmission mode of optical microscope. Therefore, the W.D. can not be characterized with our current experimental setup.

**Figure 5:**
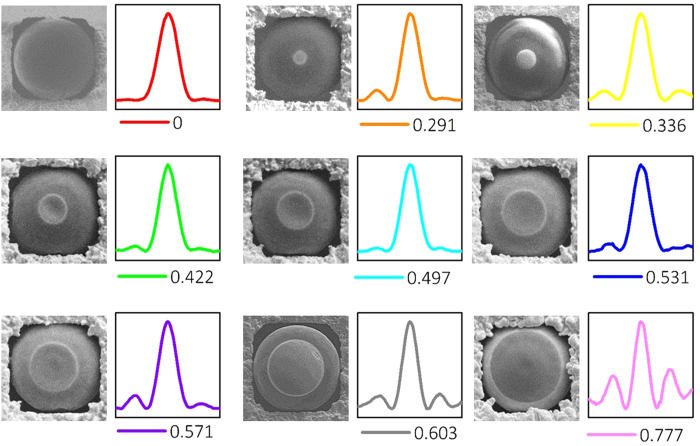
Cross-section intensity near the focal spot of microspheres (diameter of 10 *μ*m) with different cover ratios. The top-view SEM pictures show the coverage of the Pt mask. Numbers labelled under each intensity pattern indicate the cover ratio of the Pt mask. The intensity is normalized and the length of the bottom frames is 2 *μ*m.

To have a detailed comparison of the cross-section intensity distribution of the *E*_*x*_ field for different engineered microspheres, nine of the them are presented in Fig. 5. In the SEM pictures, a clear boundary between glass and Pt can be observed. The smooth edge of the deposited Pt layer minimizes the scattering of the incident beam. A thin layer of gold membrane is employed and functions as a holder to control the microsphere position during experiment. By varying the cover ratio from 0 to 0.777, the intensity distribution along the cross-section of the focal plane is presented, corresponding to each engineered microsphere design. The cross-section in vicinity of the focal plane is recorded and the intensity is normalized. In the figures, the label under each curve indicates the cover ratio and the scale of *x* axis is 2 *μ*m. It can be observed that when the cover ratio of the Pt on the microspheres increases gradually, the beam waist of the photonic nanojet is decreased. This clearly shows an effective modulation of the beam width of the photonic nanojet. When the cover ratio is higher than 0.531, an increasing sidelobe can be observed. Incident beam with high *NA*_*min*_ interferes around the focal spot and generates a Bessel-like distribution of the intensity.

## Conclusions

In summary, we design and fabricate the center-covered engineered microspheres with different cover ratios on the illumination surface. This cover layer forms a center-blocked filter which selectively transmits the incident beam into the microsphere. By varying the cover ratio of the engineered microsphere, modification of the photonic nanojet is demonstrated. When the non-transparent cover is introduced on the microsphere, the parasitic components of the incident beam are filtered out and therefore the contribution of different components to the final field are adjusted. At a wavelength of 633 nm, a sharp focal spot of 0.387 *λ* is achieved experimentally. To evaluate this modulation effect, the contribution of the incident beam at different distance from the optical axis to the photonic nanojet is analyzed. It is shown that at a larger cover ratio, the *NA*_*min*_ of the engineered microsphere is higher than that at a low cover ratio. This leads to a sharper focal spot and shorter working distance of the photonic nanojet. The modulated photonic nanojet with small beam size generated by a high cover ratio engineered microsphere is a promising approach for applications of particle acceleration, optical super-resolution imaging and optical lithography.

## Methods

### Sample fabrication

In this study, the microspheres at a diameter of 10 *μ*m are commercially available from Bangs Laboratories, Inc. The fabrication details of the gold membrane can be found in our previous study^20^. To deposit Pt layer on the microsphere surface, FEI DA 300 Focus Ion Beam (FIB) system is employed. Applying 30 KV and 50 nA of liquid metal Gallium ion sources, around 1 *μ*m thick Pt with different diameters are fabricated on the top of microspheres. During the FIB fabrication, the center of the mask pattern is aligned with the optical axis of the microsphere.

### Characterization

To characterize the photonic nanojet generated by the engineered microsphere, we design and build the experimental setup, which is schematically shown in Fig. 3a. A low power He-Ne 633 nm linear polarized laser (MellesGriot, 25-LHP-925-230) is applied as the incident light source. A half wave plate is applied to shape the linear polarization of the beam and an attenuator is employed to tune the laser intensity to a desired brightness. To manipulate the engineered microsphere in three dimensions, a thin gold membrane is made and functions as a microsphere lens holder. The thickness of the gold membrane is around 5 *μ*m and it can be observed that the hole matches the diameter of the microsphere. Thus, a strong support is provided for FIB fabrication and optical microscope characterization. During the characterization experiment, a single engineered microsphere is placed under the illumination beam. The incident beam is assumed to propagate along positive *z* axis and in parallel with the optical axis of the engineered microsphere. To record the *x* − *y* plane diffraction pattern of the photonic nanojets, a high magnification objective lens (Olympus, LMPlan Apo 150×, NA 0.9) and a high resolution CMOS camera (Nikon digital SLR camera FX-format CMOS sensor, 4908 × 3264 pixel) are used. Before the characterization, calibration of the pixel size of the CMOS camera are performed. A structure which is inspected by SEM is used as a testing sample. By calibrating the lengths in SEM and optical images, the size of each pixel of the CMOS camera is obtained. The cross section of the photonic nanojet is captured in the vicinity of the focal plane. For each cover ratio, three engineered microspheres are fabricated. The final results are summarized based on multiple times of experimental confirmation.

## Additional Information

**How to cite this article**: Wu, M. *et al*. Super-focusing of center-covered engineered microsphere. *Sci. Rep.* **6**, 31637; doi: 10.1038/srep31637 (2016).
